# Identification and trichothecene genotypes of *Fusarium graminearum* species complex from wheat in Taiwan

**DOI:** 10.1186/s40529-016-0156-4

**Published:** 2017-01-02

**Authors:** Chih-Li Wang, Yi-Hong Cheng

**Affiliations:** Department of Plant Pathology, National Chung Hsing University, Taichung, 40227 Taiwan

**Keywords:** Fusarium head blight, *Fusarium asiaticum*, *Fusarium graminearum*, *Fusarium meridionale*, Deoxynivalenol, Nivalenol

## Abstract

**Background:**

Fusarium head blight (FHB) of wheat caused by *Fusarium graminearum* species complex (FGSC) is a devastating disease worldwide. The pathogens not only reduce the yield of wheat, but also impact the quality of wheat by contamination with trichothecene mycotoxins. A systematic investigation on the pathogens of FHB in Taiwan is lacking. Here, molecular and morphological approaches were used to identify species of the Taiwanese FGSC isolates and determine their trichothecene genotypes.

**Results:**

In this study, a total of 195 isolates of FGSC from diseased wheat were collected from 8 areas of northern and central Taiwan. All isolates were subjected to seedling inoculation for verification of pathogenicity. The pathogenic isolates were genetically characterized by sequence characterized amplified region (SCAR), PCR- restriction fragment length polymorphism (RFLP), phylogenetic analysis and fixed nucleotides to clarify their phylogenetic species, and by PCR assays of *TRI* genes to determine trichothecene genotypes. They were identified as *F. asiaticum*, *F. graminearum* sensu stricto, *F. meridionale* and an unknown species. Isolates of *F. asiaticum* were the major causal agents (98%) in this investigated population and were comprised of SCAR type 5 (75%), SCAR type 4 (21%) and SCAR type 3 (2%). Their trichothecene genotypes were either 15-acetyl-deoxynivalenol (15-ADON) (83%) or nivalenol (NIV) genotype (17%). These genetic characterizations indicated that *F. asiaticum* (15-ADON SCAR type 5) accounts for 60% of this Taiwanese population. Virulence assay on wheat heads indicated virulence of *F. asiaticum* isolates in subpopulations divided by SCAR types or trichothecene genotypes were comparable, suggesting other factors influence the unequal subpopulation sizes.

**Conclusions:**

This is the first study that FGSC isolates in Taiwan were systematically collected and characterized. In addition to *F. graminearum* sensu stricto and *F. meridionale*, *F. asiaticum* with 15-ADON genotype was identified as the predominate species in Taiwan. In contrast to Chinese and Japanese populations that *F. asiaticum* isolates were typically of 3-ADON or NIV genotype, the predominate 15-ADON genotype in Taiwanese population was unique among *F. asiaticum* populations and represented the southernmost 15-ADON genotype population in East Asia.

**Electronic supplementary material:**

The online version of this article (doi:10.1186/s40529-016-0156-4) contains supplementary material, which is available to authorized users.

## Background

Wheat was an important crop that grew in many counties of Taiwan in 1960s. Due to the impact of world trade, wheat production and plantation areas have shrunk dramatically over the years. There were only limited areas in central Taiwan growing wheat for malt production in the past 40 years. In recent years, the acreages of wheat were gradually increased from central Taiwan to southern and northern Taiwan because of the advocacy of eating locally grown crops and a governmental policy of raising food self-sufficiency rates. In an informal survey, Fusarium head blight (FHB) was found to be prevalent in many counties after the anthesis stage of wheat (Kuo et al. 2014).

FHB could be caused by a number of *Fusarium* species. Some species played a more important role than others, depending on geographic regions. Among them, *F. graminearum* sensu lato was reported as an economically devastating pathogen (Windels 2000). The morphologically characterized *F. graminearum* sensu lato was revealed as a species complex since 2000 (O’Donnell et al. 2000). To date, at least 15 species were contained within *F. graminearum* species complex (FGSC), isolated from various hosts and substrates that have been established based on the genealogical concordance phylogenetic species recognition (O’Donnell et al. 2004, 2008; Sarver et al. 2011; Starkey et al. 2007; Yli-Mattila et al. 2009). Except for *F. graminearum* sensu stricto (s. str.) that distributed worldwide, most species of FGSC were mainly restricted to few geographic regions (O’Donnell et al. 2004). *Fusarium austroamericanum*, for example, was distributed in South America. *Fusarium asiaticum* was reported in Brazil, USA, and mainly in Asian countries. In spite of numerous causal agents of FHB to wheat, particular species usually were more prevalent than others. For instance, among 623 isolates of FHB pathogens from 7 states of United States, 523 isolates belonged to *F. graminearum* s. str. (Zeller et al. 2004). *Fusarium graminearum* s. str. represented 76% of a genotyped Louisiana population (154 isolates) (Gale et al. 2011). These studies illustrated that *F. graminearum* s. str. was the predominate species in the United States. *Fusarium asiaticum* (77%) and *F. graminearum* s. str. (23%) were identified from 299 isolates collected from 12 provinces in China that were mainly located in the Yangtze River valleys (Zhang et al. 2007). Among 530 isolates collected from 6 provinces (Jiangsu, Anhui, Henan, Hebei, Shandong and Hubei) in China, 65% were *F. asiaticum* and 34% were *F. graminearum* s. str. (Shen et al. 2012). In a broader sampling from 15 provinces in China, *F. graminearum* s. str. (36%) and *F. asiaticum* (58%) were the major FHB pathogens of 469 isolates though the other 6 *Fusarium* species were also found (Zhang et al. 2012). From 35 prefectures in Japan, 246 *F. asiaticum* and 50 *F. graminearum* s. str. were identified in 298 collected isolates (Suga et al. 2008). In South Korea, of 356 isolates from rice grown in 5 provinces, 333 were *F. asiaticum* and 23 were *F. graminearum* s. str. (Lee et al. 2009). Of 568 isolates from maize at eight locations in South Korea, *F. graminearum* s. str. (75%) was most common species followed by *F. asiaticum* (12%) (Lee et al. 2012). Above information also indicated that *F. asiaticum* and *F. graminearum* were the major FHB pathogens on wheat in China and Japan, and the major FGSC species in South Korea.

In addition to yield loss, diseased grains of FHB usually accumulated trichothecenes that were associated with toxicoses in humans and farm animals (Desjardins 2006). Species of FGSC were trichothecene B-producing fungi that could be classified into three strain-specific chemotypes based on their trichothecene production: the 3-ADON chemotype isolates produced deoxynivalenol (DON) and 3-acetyl-deoxynivalenol (3-ADON); the 15-ADON chemotype isolates produced DON and 15-acetyl-deoxynivalenol (15-ADON); and the NIV chemotype isolates produced nivalenol (NIV) and 4-acetylnivalenol (Ward et al. 2002; Miller et al. 1991). Individual trichothecene may differ in toxicological effects on plants and animals. DON inhibited root growth and coleoptiles elongation of wheat, but NIV had no effect at similar concentration (Eudes et al. 2000; Shimada and Otani 1990). DON was shown to be a virulent factor of FHB to wheat whereas NIV was a virulent factor of ear rot to maize (Maier et al. 2006; Jansen et al. 2005; Proctor et al. 1995). Compared to DON, some studies observed higher cytotoxicity for NIV (Minervini et al. 2004). These differences in toxic effects made chemotypes of isolates important in geographic distribution and population composition of fungal pathogens.

The conventional method for chemotyping the trichothecenes of *Fusarium* species was the HPLC or GC/MS analysis of culture extracts from individual isolates. PCR methods for genotyping the trichothecene chemotypes of *Fusarium* species were developed according to allelic variants of trichothecene biosynthesis genes in isolates with strain-specific chemotypes. Sequences of *TRI7*, *TRI12* and *TRI13* genes, for instances, were distinct in DON- and NIV-producing isolates (Lee et al. 2001; Chandler et al. 2003; Ward et al. 2002; Quarta et al. 2006). Primers designed on *TRI3* and *TRI12* were used to assess 3-ADON- and 15-ADON-producing isolates (Jennings et al. 2004; Quarta et al. 2006; Ward et al. 2002). These studies also demonstrated that results of genotyping were highly correlated to these of chemical chemotyping. The trichothecene genotyping by PCR assays allowed rapid screening of a large number of isolates, and identification of the increasing occurrence of high toxigenic population such as 3-ADON- and NIV-producing isolates (Pasquali et al. 2010; Puri and Zhong 2010; Zhang et al. 2012).

In Taiwan, FHB of wheat caused by *F. graminearum* sensu lato was first reported in 1919 (Sawada 1919). Although other species of potential FHB pathogens such as *F. poae*, *F. avenaceum* and *F. culmorum* were also reported, none of them were isolated from wheat (Huang and Sun 1997). These earlier studies were based on the morphological characterization which was not able to resolve species in a species complex. Along with the significant occurrence of FHB, there was an increasing need to clarify the composition of the causal agents, contributing to the formulation of control strategies and for evaluation of mycotoxin threat. Trichothecenes, especially the DON (also known as vomitoxin), produced in the infected grains will become a concern for consumers. Unfortunately, there was no systematic survey carried out regarding these concerns in Taiwan. This study was aimed to identify the species of FHB pathogens including species of FGSC, and to determine their trichothecene genotypes. Results will also improve our understanding of the distribution and trichothecene genotypes of FGSC species in wheat production areas in East Asia.

## Methods

### Strain isolation and morphological identification

Wheat samples were collected from 10 areas of 6 cities and counties from northern Taiwan to southern Taiwan. Potential Fusarium head blight pathogens of wheat were isolated from seeds and wheat heads showing head blight symptoms. In areas of southern Taiwan where Fusarium head blight was not observed, around 100 wheat heads were randomly collected from each field for fungal isolation. For isolating fungi from diseased or symptomless wheat heads, 10 every other kernels were picked from bottom to top of a head, and incubated on 2% (w/v) water agar (WA) at room temperature for 3–5 days. After fungi grew out, one isolate was picked from each head for further study. Therefore, all isolates were obtained from different plants. A modified blotter method (De Tempe 1953) was used for fungal isolation from seeds. Briefly, seeds were treated with 1% sodium chloride for 1 min, rinsed with sterilized water for 3 times, and then incubated on a piece of wet autoclaved filter paper in 9-cm Petri dishes at 24 °C for 7 days. Ten seeds were plated in each plate, and 400 seeds were randomly sampled from each bag of seeds. Fungal colonies secreting carmine red pigment in WA or filter paper and producing characterized spindle-sickle macroconidia were selected for further purification. Single macroconidium of *Fusarium* was isolated for analyses. For morphological analysis, *Fusarium* isolates were grown on potato dextrose agar (PDA; Difco, NJ, USA) to observe culture characteristics and carnation leaf agar (CLA) to examine the morphology of macroconidia (Leslie and Summerell 2006). Thirty macroconidia with 4–5 septa from each isolate were measured to determine the conidial size. Perithecia were induced on carrot medium (5 g of Bacto agar, 400 g freshly sliced carrots, per liter of distilled water) (Cavinder et al. 2012). Ascospores released from asci were measured and characterized.

### Molecular identification on FGSC

For genomic DNA isolation, fungal isolates were grown in yeast extract peptone dextrose broth (0.3 g of yeast extract, 1.0 g of peptone and 1.0 g of dextrose in 100 ml of distilled water) at 25 °C for 3 days. Fungal mycelia were harvested with miracloth (pore size: 22–25 µm) (Calbiochem, CA, USA) and DNAs were extracted following the standard procedures (Sambrook and Russell 2001). Primers Fg16F (5′-CTCCG-GATATGTTGCGTCAA-3′) and Fg16R (5′-GGTAGGTATCCGACATGGCAA-3′) were used as a pair of specific PCR primers to determine if a *Fusarium* isolate belongs to a FGSC isolate, and sizes of this PCR amplicons were used to assign the sequence characterized amplified region (SCAR) types to each isolate (Carter et al. 2000; Nicholson et al. 1998; Carter et al. 2002). The PCR program was set according to Nicolson et al. (1998). Except where indicated otherwise all PCR reactions in this study were carried out in a total volume of 25 µl containing 19.7 µl of MilliQ water, 2.5 µl of 10× buffer, 0.5 µl of dNTP (200 µM each of dATP, dTTP, dGTP and dCTP), 0.5 µl of each primer (10 µM), 1 µl of DNA template (100 ng/µl), and 0.3 µl of DNA polymerase (Pro Taq plus; Protech Technology Enterprise, Taipei, Taiwan). PCR amplification was conducted in a thermal cycler (Applied Biosystems 2720; Life Technologies, CA, USA). Electrophoresis was performed in 1.5% (w/v) TAE agarose gel, and ethidium bromide was used for gel staining. PCR-restriction fragment length polymorphism (PCR–RFLP) of partial histone gene was applied to identify *F. graminearum* s. str. and *F. asiaticum*. The partial histone H3 gene fragment with size of 223 bp was amplified using primers H3dStyI (5′-AGCATCACCYGAACATCGCATCATCCCATG-3′) and H3R1 (5′-TTGGACTGG-ATRGTAACACGC-3′), and purified with Plus DNA Clean/Extraction Kit (GeneMark, Taichung, Taiwan). The purified PCR products were digested with *Eco*RV and *Sty*I, respectively (Suga et al. 2008). The digestion results were visualized by electrophoresis in 2.5% (w/v) TAE (Tris-acetate-EDTA) agarose gel.

### Trichothecene genotyping

Primers locating at *TRI13* gene were used to differentiate the genotypes of DON or NIV. Primers Tri13F (5′-CATCATGAGACTTGTKCRAGTTTGGG-3′) and Tri13DONR (5′-GCTAGATCGATTGTTGCATTGAG-3′) would amplify a PCR band from DON genotype isolates, but not from NIV genotype isolates. In contrast, primers Tri13NIVF (5′-CCAAATCCGAAAACCGCAG-3′) and Tri13R (5′-TTGAAAGCTCCAATGTCGTG-3′) would amplify a band from NIV genotype isolates, but not from DON genotype isolates (Chandler et al. 2003). Primers Tri303F (5′-GATGGCCGCAAGTGGA-3′) and Tri303R (5′-GCCGGACTGCCCTATTG-3′) were used to detect 3-ADON genotype whereas primers Tri315F (5′-CTCGCTGAAGTTGGACGTAA-3′) and Tri315R (5′-GTCTATGCTCTCAACGGACAAC-3′) were used to determine 15-ADON genotype (Jennings et al. 2004).

### Pathogenicity assays

To induce the production of conidia, agar blocks containing mycelia of each isolate were cultured in mung bean broth (40 g of mung beans was steeped in 1 l of just boiled distilled water for 10 min. After beans were removed by cheesecloth, the filtrate was autoclaved for use.) at 25 °C and 100 rpm for 3 days (Wu et al. 2005). Conidia were filtrated through miracloth and counted by hemocytometer. The suspension was centrifuged and adjusted to 10^5^ conidia/ml with sterilized water and used for pathogenicity tests. The local wheat cultivar, Taichung Sel. 2, was used as host plants. A seedling coleoptile inoculation method (Wu et al. 2005) was modified to quickly determine the pathogenicity of all isolates. Briefly, seedling coleoptiles were wounded by a needle, and then immersing in a conidia suspension (10^5^ conidia/ml) for 30 min. Five seedlings were inoculated with each isolate. Inoculated seedlings were placed on wet paper towel in Petri dishes and put in moister chambers at 24 °C with 12 h light cycle. To evaluate the virulence of each isolate, 5 µl conidia suspension (10^5^ conidia/ml) was inoculated at the interior of the two central spikelets of each head at the anthesis stage (Malbrán et al. 2012). The inoculated wheat heads were covered with plastic bags for 48 h to maintain moisture. Three replicates were conducted for each isolate. Virulence was evaluated by the number of symptomatic spikelets at the 12 days post inoculation (dpi).

### Phylogenetic analysis

The partial sequences of *RED* gene and *TEF*-*1α* gene of 19 selected representative isolates were used for resolving their phylogenetic relationships with reference strains of all known species of FGSC. A PCR product of *RED* sequence in a range from 900 to 1000 bp was amplified with primers RED1d (5′- TCTCAGAAAGACGCATATATG-3′) and RED2 (5′- CGTAACTGCGTCATTCGGC-3′). *TEF*-*1α* sequence in a range from 600 to 700 bp was amplified with primers TEF1 (5′- ATGGGTAAGGAGGACAAGAC-3′) and TEF2 (5′- GGAAGTACCAGTGATCATGTT-3′) (O’Donnell et al. 2000). PCR products were purified for sequencing with the forward and reverse primers. Phylogenetic analysis was performed by MEGA 6 (Tamura et al. 2013). ClustalW software was applied to align the two gene sequences from the 19 isolates in this study and other reference strains belonging to species of FGSC. The concatenate sequences of *RED* and *TEF*-*1α* genes were analyzed by the maximum likelihood method with 1000 bootstrap replications.

## Results

### Species diagnosis and SCAR types of FGSC isolates from wheat

**Fig. 1:**
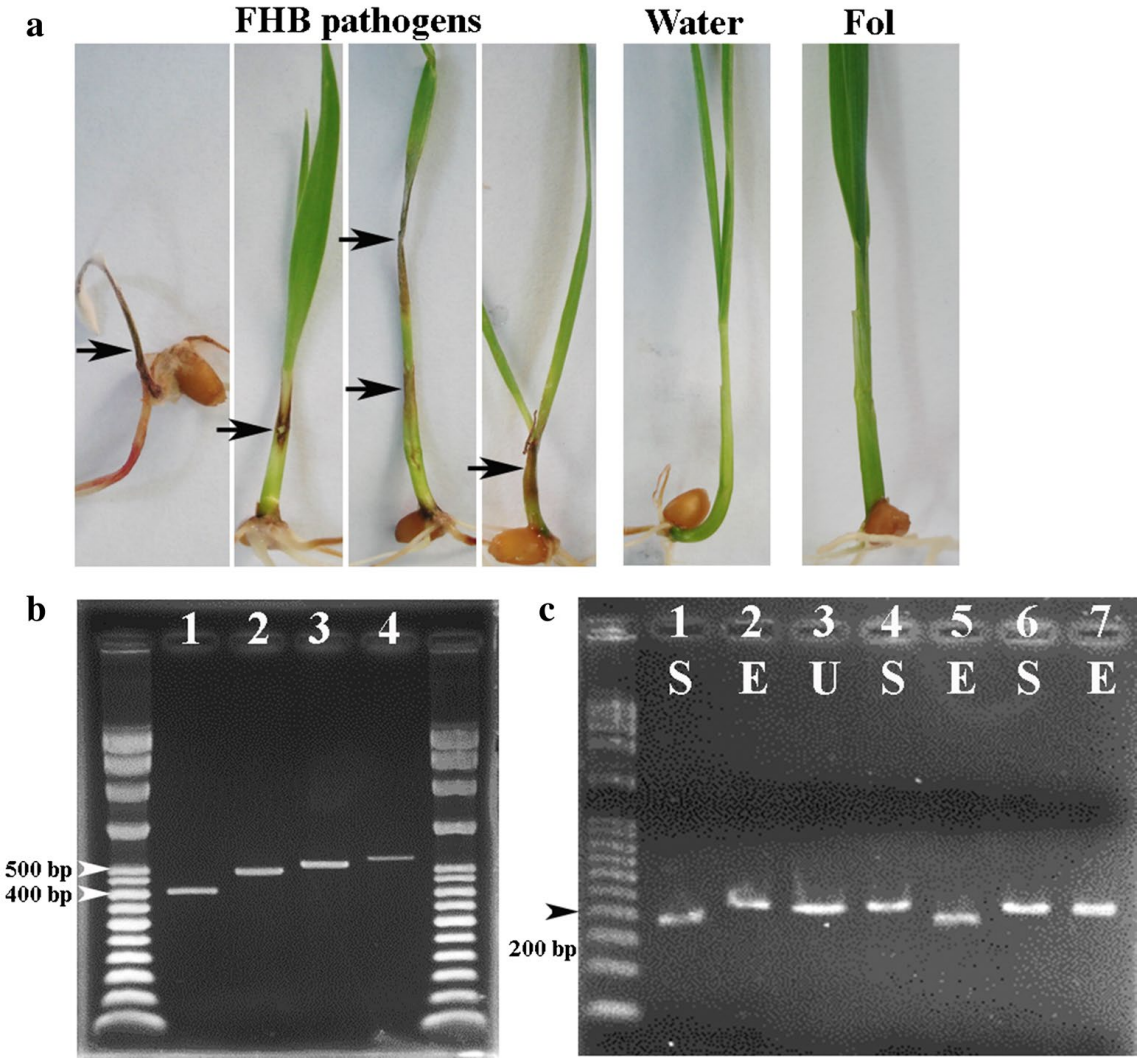
Representative results of seedling pathogenicity assays, SCAR types, and PCR–RFLP diagnosis. **a** The pathogenicity assay with seedling coleoptile inoculation method was used to test if all isolates are pathogenic to wheat. Necrotic leaf sheath lesions (*arrows*) indicated that the tested isolates were FHB pathogens. No symptom displayed after inoculation with sterilized water or *Fusarium oxysporum* f.sp. *lycopersici* (Fol, not a FHB pathogen), respectively. **b** The variation of SCAR types in studied population. The* first lane* is the DNA ladder; *Lane 1* is SCAR type 1 (410 bp); *Lane 2* is SCAR type 2 or type 5 (497 bp); *Lane 3* is SCAR type 3 (527 bp); *Lane 4* is SCAR type 4 (557 bp). The* last lane* is another DNA ladder. **c** A PCR–RFLP method for diagnosis of *F. asiaticum* and *F. graminearum* s. str. through a partial histone H3 fragment. A *F. asiaticum* isolate will be digested by *Sty*I but not *Eco*RV whereas a *F. graminearum* s. str. isolate will be digested by *Eco*RV but not *Sty*I. Other species will not be digested by the two enzymes. The* first lane* is the DNA ladder; *Lanes 1* to *3* are a *F. asiaticum* isolate (Pingzhen1-49); *Lanes 4* and *5* are a *F. graminearum* s. str. isolate (Daya211-13);* Lanes 6 and 7* are an unknown species isolate (Daya272-3). Different restriction enzyme treatments are indicated: for “S” this is *Sty*I, for “E” this is *Eco*RV, and for “U” this is the uncut control

Based on secretion of carmine red pigment and sickle-shaped macroconidia of *Fusarium*, a total of 203 potential causal agents of Fusarium head blight were isolated. Almost 100% of fungi that produce the feature pigment and macroconidia could be isolated from wheat heads with typical symptom of FHB from most areas such as Daya, Pingzhen, Guanying and Xinwu where the occurrence of FHB was more frequent than other areas. In areas of southern Taiwan (Chiayi county and Tainan city), the FHB was not observed. Wheat heads with suspicious symptoms were collected for isolation. In this case, the isolation rate of carmine red pigment colony was rare. A seedling coleoptile inoculation method was adapted to quickly assay the pathogenicity of each isolate. Pathogenic isolates would cause necrotic lesions on leaf sheath or leaf blade while the water control or non-pathogenic *Fusarium* isolates to wheat such as the *Fusarium oxysporum* f. sp. *lycopersici* did not produce any lesion on wheat seedlings (Fig. 1a). Among them, 195 isolates (186 isolates from diseased plants and 9 isolates from seeds) collected from 8 areas of northern and central Taiwan were determined as wheat pathogens whereas 8 isolates obtained from 600 wheat heads with suspicious symptoms produced in southern Taiwan (Chiayi county and Tainan city) were not FHB pathogens. Of these 195 isolates, 193 isolates but 2 isolates (Dacheng 1-2 and Dacheng 2-1) showed the positive band in the PCR results using primers Fg16F and Fg16R, indicating that most of them belonged to FGSC isolates. Different sizes of the PCR bands were isolated and cloned for sequencing to determine the exact number of nucleotide base pairs (Fig. 1b). The 193 isolates were assigned into different SCAR types based on their band sizes. The SCAR type 5 (497 bp) isolates (75%) were most prevalent in population. The SCAR type 4 (557 bp) isolates represented 21% of the population. The SCAR type 3 (527 bp) isolates was only 1.5% of this studied population. Both SCAR type 1 and SCAR type 2 were found only in one isolate. Isolates from Daya area (Taichung city, central Taiwan) were most diverse in SCAR types. Isolates of SCAR type 5 and SCAR type 4 consistently presented in 3- 3.5–1 ratio in most geographic areas (Table 1). A PCR–RFLP method that was based on fixed nucleotide character states specific to *F. graminearum* s. str. and *F. asiaticum* has been reliable to diagnose 296 isolates of the two species in Japan (Suga et al. 2008), 530 isolates of the two species in China (Shen et al. 2012), and 41 isolates of *F. asiaticum* in US (Gale et al. 2011). Since *F. graminearum* s. str. and *F. asiaticum* were the most common species of FGSC in Asian countries (e.g. China, South Korea and Japan) (review see van der Lee et al. 2015; Wang et al. 2011), we used the method to diagnose if any of these Taiwanese FGSC isolates belong to the two species. In an earlier report, the partial histone H3 gene fragment of *F. asiaticum* would be cut into 191 and 32 bp by *Sty*I whereas the partial histone H3 gene fragment of *F. graminearum* s. str. would be cut into 195 and 28 bp by *Eco*RV (Suga et al. 2008). The band shifted to a reduced size, after the restriction enzyme treatments, indicating a positive digestion (Fig. 1c). DNAs of a total of 192 out of the 195 isolates were digested into smaller fragments by *Sty*I, suggesting that *F. asiaticum* (99%) was the predominate species of FGSC in Taiwan. Only one isolate (Daya211-13) was diagnosed as *F. graminearum* s. str. Two isolates (Fanyuan1-11 and Daya272-3) that failed to be digested by neither *Sty*I nor *Eco*RV were selected for further identification by phylogenetic analysis.

**Table 1:** The geographic distribution of identified species and SCAR types of all isolates

Collection area	No. of isolates	*F. asiaticum*			*F. graminearum* s. str.		*F. meridionale*		Unknown species	
		Type^a^ 3	Type 4	Type 5	Type 1		Type 5		Type 2	
Northern Taiwan										
Taoyuan city										
Guanying	30		7	23						
Xinwu	30		8	22						
Pinzhen	30		3	27						
Central Taiwan										
Miaoli county										
Yuanli	3		1	2						
Taichung city										
Daya	70	3	15	50		1				1
Waipu	4			4						
Changhua county										
Fanyuan	23		5	17				1		
Dacheng	5^b^		2	1						
Total	195^b^	3	41	146		1		1		1

^a^The SCAR types were defined by Carter et al. (2002). Type 1 is 410 bp; Type 2 is 479 bp; Type 3 is 527 bp; Type 4 is 557 bp; Type 5 is 479 bp. Type 2 and Type 5 are different in nucleotide sequences

^b^The SCAR patterns were not amplified from two *F. asiaticum* isolates (Dacheng 1-2 and Dacheng 2-1)

### Phylogenetic analysis

**Fig. 2:**
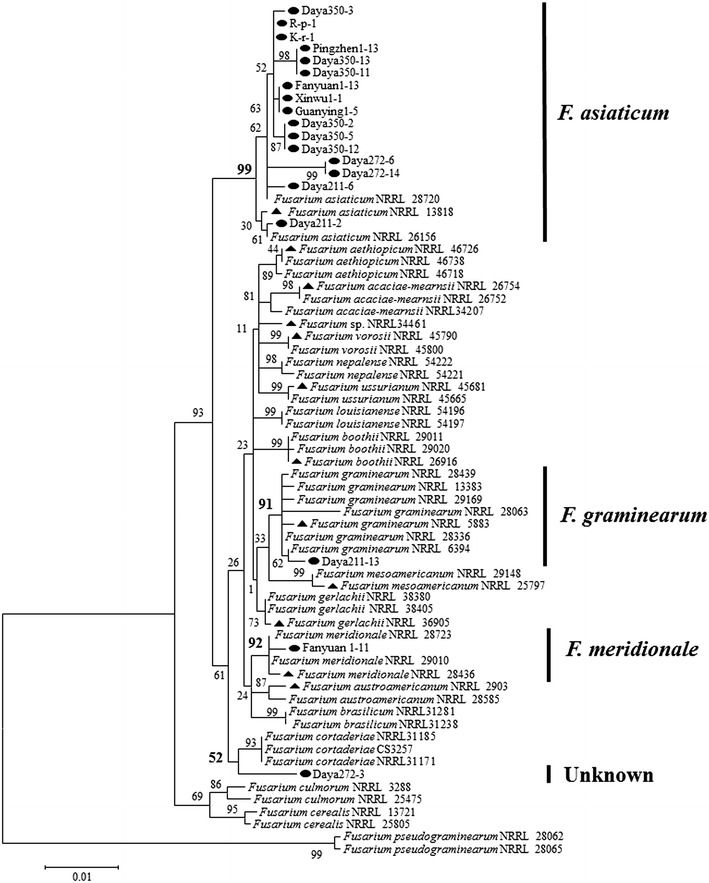
The phylogenetic analysis. The phylogenetic tree of representative strains of FHB pathogens in Taiwan based on partial sequences of *RED* and *TEF*-*1α* genes. The maximum likelihood method was used for the analysis with 1000 bootstrap repeats (*black circle* isolates in this study, *filled triangle* type strains of species of FGSC)

To further confirm the diagnostic results of PCR–RFLP and determine the 2 unidentified isolates, a total of 19 representative isolates including 16 isolates of *F. asiaticum*, 1 isolate of *F. graminearum* s. str., and the 2 unidentified isolates were further investigated for their phylogenetic relationships with reference strains and type strains of all known FGSC species. The 16 isolates of *F. asiaticum* were randomly selected from each SCAR type and trichothecene genotype. For phylogenetic analysis, *RED* gene and *TEF*-*1α* gene of the 19 representative isolates were sequenced and deposited at GenBank (http://www.ncbi.nlm.nih.gov/genbank/) with accession numbers (Table 2). A total of 1296 informative nucleotides were used for analysis including 775 nucleotides of *RED* gene and 521 nucleotides of *TEF*-*1α* gene. The 16 isolates of *F. asiaticum* were well clustered with 3 reference *F. asiaticum* strains with a high bootstrap value (99%). The *F. graminearum* s. str. isolate was also placed in the *F. graminearum* s. str. clade with a bootstrap value of 91%. These results not only confirmed the PCR–RFLP method was reliable, but also illustrated this phylogenetic tree was able to resolve the phylogenetic relationship at species level to some extent. One of the unidentified isolates (Fanyuan1-11) was clearly clustered with 2 reference strains and the type strain of *F. meridionale* with a bootstrap value of 92%. The other unidentified isolate (Daya272-3) was closest to the *F. cortaderiae* clade, but had a low bootstrap value of 52% and this did not support the relationship (Fig. 2).

**Table 2:** The list of representative isolates used for phylogenetic analysis

Species of FGSC and isolate	SCAR type^a^	Trichothecene genotype	Accession number	
			Reductase	TEF-1α
*F. asiaticum*				
Daya350-5	Type 5	NIV	KT372157	KT380119
Daya211-2	Type 5	NIV	KT372158	KT380120
Fanyuan1-13	Type 5	NIV	KT372159	KT380121
R-p-1	Type 5	15-ADON	KT372149	KT380111
Daya350-3	Type 5	15-ADON	KT372162	KT380124
Daya211-6	Type 5	15-ADON	KT372164	KT380126
Xinwu1-1	Type 5	15-ADON	KT372163	KT380125
Daya350-11	Type 3	15-ADON	KT372147	KT380109
Daya350-12	Type 3	15-ADON	KT372152	KT380114
Daya272-14	Type 3	15-ADON	KT372153	KT380115
Daya350-2	Type 4	NIV	KT372154	KT380116
Daya272-6	Type 4	NIV	KT372155	KT380117
Pingzhen1-13	Type 4	NIV	KT372156	KT380118
Daya350-13	Type 4	15-ADON	KT372160	KT380122
k-r-1	Type 4	15-ADON	KT372150	KT380112
Guanying 1-5	Type 4	15-ADON	KT372161	KT380123
*F. graminearum* s. str.				
Daya211-13	Type 1	15-ADON	KT334553	KT380127
*F. meridionale*				
Fanyuan1-11	Type 5	NIV	KT372148	KT380110
Unknown species				
Daya272-3	Type 2	NIV	KT372151	KT380113

^a^The SCAR types were defined by Carter et al. (2002). Type 1 is 410 bp; Type 2 is 479 bp; Type 3 is 527 bp; Type 4 is 557 bp; Type 5 is 479 bp. Type 2 and Type 5 are different in nucleotide sequences

All sequenced isolates of identified *F. asiaticum* were further compared with the species type strain NRRL 13818 on the defined fixed nucleotides of *RED* sequence, and the results showed that they were identical at all fixed nucleotides. The *RED* and *TEF*-*1α* sequences of identified *F. graminearum* s. str. and *F. meridionale* were aligned with reference strains *F. graminearum* s. str. NRRL 5883 and *F. meridionale* NRRL 28436, respectively. All fixed nucleotides were also conserved in the investigated strains in the 2 gene alignments. In addition, morphology of all sequenced isolates was characterized and was indistinguishable from each other. All isolates produced carmine red pigment on PDA and CLA, and formed macroconidia and no microconidia. Macroconidia from CLA were gradually curved and 52–63 × 5–6 μm with 5–6 septa. All isolates were homothallic and produced various numbers of dark blue perithecia containing a number of asci on carrot medium. Each ascus bore 8 ascospores. Ascospores were 21–24 × 3–5 μm with 1–3 septa, hyaline in color and fusiform with rounded ends (Additional file 1: Figure S1). These morphological characteristics conformed to the defined characteristics of FGSC.

### Trichothecene genotypes

**Fig. 3:**
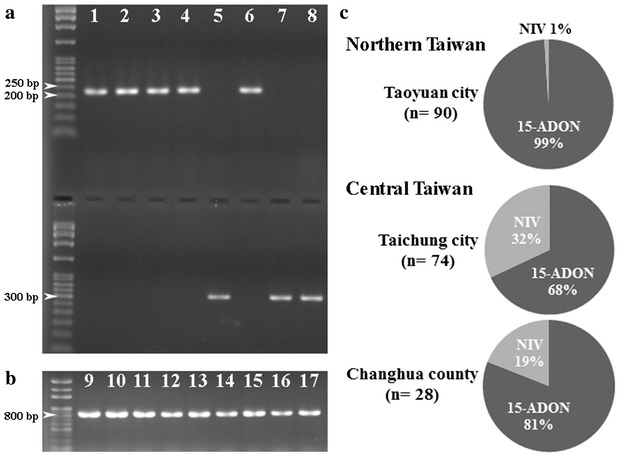
The identification and distribution of trichothecene genotypes of FGSC isolates. **a** Identification of DON or NIV genotype by the amplification of *TRI13* gene with primers Tri13F and Tri13DONR (*upper panel*), and primers Tri13NIVF and Tri13R (*lower panel*), respectively. The* first lane* is the DNA ladder; *Lane 1* is Daya 350-12; *Lane 2* is Daya 350-13; *Lane 3* is Daya 350-14; *Lane 4* is Daya 350-15; *Lane 5* is Daya 350-16; *Lane 6* is Daya 211-1; *Lane 7* is Daya 211-2; *Lane 8* is Daya 211-3. **b** Identification of 15-ADON genotype by the amplification of *TRI3* gene with primers Tri315F and Tri315R. The* first lane* is the DNA ladder; *Lane 9* is Daya 350-11; *Lane 10* is Daya 350-12; *Lane 11* is Daya 350-13; *Lane 12* is Daya 211-6; *Lane 13* is Xinwu 1-1; *Lane 14* is Pingzhen1-13; *Lane 15* is R-P-1; *Lane 16* is Guanying 1-5; *Lane 17* is Daya 211-13. **c** Distributions of FGSC isolates regarding trichothecene genotypes in northern (Taoyuan city) and central (Taichung city and Changhua county) Taiwan

The allelic nucleotide polymorphism of *TRI13* gene was used to distinguish DON genotype or NIV genotype. A PCR band of about 227 bp indicating DON genotype was amplified from 162 isolates (83%) by primers Tri13F and Tri13DONR. On the other hand, a PCR band of about 312 bp indicating NIV genotype was amplified from 33 isolates (17%) by primers Tri13NIVF and Tri13R (Fig. 3a). The DON genotype isolates were further differentiated as 3-ADON or 15-ADON isolates by primers designed from the different alleles of *TRI3* gene. All DON genotype isolates generated a 864 bp band in the PCR reactions with primers Tri315F and Tri315R, but not a 586 bp band with primers Tri303F and Tri303R, indicating that all DON genotype isolates were of 15-ADON genotype (Fig. 3b). In northern Taiwan, all isolates but one belonged to 15-ADON (99%). In central Taiwan, NIV isolates accounted for 32 and 19% of populations in Taichung city and Changhua county, respectively (Fig. 3c). As with SCAR types, trichothecene genotypes were more diverse in central Taiwan than northern Taiwan.

Combined with the above results of isolate identity and SCAR types, one would conclude that the most predominate species and genotype is *F. asiaticum* 15-ADON SCAR type 5 (60%). *Fusarium asiaticum* 15-ADON SCAR type 4 isolates and *F. asiaticum* NIV SCAR type 5 isolates contributed 19 and 15% of the studied population, respectively. The single *F. graminearum* s. str. isolate was of 15-ADON genotype whereas the single *F. meridionale* isolate and the unknown species isolate were of NIV genotype (Table 3).

**Table 3:** The trichothecene genotypes of all species with reference to SCAR types

Genotype	No. of isolates	*F. asiaticum*			*F. graminearum* s.s.	*F. meridionale*	Unknown
		Type 5^a^	Type 3	Type 4	Type 1	Type 5	Type 2
DON	162^b^						
15-ADON	162^b^	118 (60.5%)	3 (1.5%)	38 (19.4%)	1 (0.5%)		
NIV	33	30 (15.3%)		3 (1.5%)		1 (0.5%)	1 (0.5%)
Total	195^b^	146	3	41	1	1	1

^a^The SCAR types were defined by Carter et al. (2002). Type 1 is 410 bp; Type 2 is 479 bp; Type 3 is 527 bp; Type 4 is 557 bp; Type 5 is 479 bp. Type 2 and Type 5 are different in nucleotide sequences

^b^The SCAR types of two *F. asiaticum* isolates (Dacheng 1-2 and Dacheng 2-1) were not available

### Virulence comparison among isolates with different SCAR types or trichothecene genotypes

**Fig. 4:**
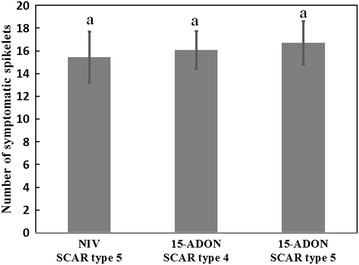
The virulence comparison of *F. asiaticum* isolates with different genotypes or SCAR types. The pathogenicity assays were performed by point inoculation on central spikelets of wheat heads. Data were analyzed by LSD method with the Software SPSS

To determine if virulence variation could account for the unequal subpopulation sizes of *F. asiaticum* 15-ADON SCAR type 5 isolates (60%), *F. asiaticum* 15-ADON SCAR type 4 isolates (19%) and *F. asiaticum* NIV SCAR type 5 isolates (15%), 10 isolates from each of the 3 subpopulations were randomly selected to compare their virulence on wheat heads. Conidial suspension of each isolate was inoculated on the 2 central spikelets of heads. Number of the discolored spikelet was counted to evaluate the virulence of each isolate at the 12 dpi. Although isolates of the *F. asiaticum* 15-ADON SCAR type 5 subpopulation caused slightly more symptomatic spikelets in average than isolates in the other two subpopulations, the differences did not statistically distinguish them in virulence to wheat (Fig. 4). This result suggests that the difference of trichothecene genotypes (15-ADON and NIV) or SCAR types (SCAR type 4 and SCAR type 5) did not explain the unequal subpopulation sizes.

## Discussion

Fungal isolates collected in this study were from areas of northern and central Taiwan located from North 23°50′ to North 25°02′ which represented one of the wheat production areas at the lowest latitude in the world. FHB pathogens were not detected from samples collected from southern Taiwan near or lower than the Tropic of Cancer. It would be interesting to dissect the reasons of no detection in southern Taiwan, including the presence or absence of pathogens, pathogen survival ability in high temperature, alternative hosts and environmental factors for disease development. In this study, all pathogenic isolates were considered to be members of FGSC by PCR amplification with primers Fg16F and Fg16R and morphological characteristics of asexual and sexual stages. Because species of FGSC were established based on the molecular characteristics and cannot be identified through the morphological characteristics (O’Donnell et al. 2004; Sarver et al. 2011), we used molecular methods to determine the species of each isolate. As mentioned earlier, *F. asiaticum* and *F. graminearum* s. str. have been reported as predominate species of FGSC in China and Japan (Shen et al. 2012; Zhang et al. 2007; Suga et al. 2008; Zhang et al. 2012). A PCR–RFLP method has been reliable to diagnose 867 isolates of *F. graminearum* s. str. and *F. asiaticum* in Japan, China and US (Shen et al. 2012; Gale et al. 2011; Suga et al. 2008). We, therefore, applied the PCR–RFLP diagnosis method to detect *F. asiaticum* and *F. graminearum* s. str. from collected isolates. A phylogenetic analysis based on *RED* and *TEF*-*1α* sequences complied with the diagnosed results, and further identified a *F. meridionale* isolate in our collection. As shown in studies, *RED* and *TEF*-*1α* sequences were able to separate most species of FGSC and used at the initial stage to discover a novel species (O’Donnell et al. 2004; Starkey et al. 2007). These identifications of *F. asiaticum*, *F. graminearum* s. str. and *F. meridionale* were also supported by the fixed nucleotides presenting in the 2 gene sequences and by the morphology of the sexual and conidial stages. Nevertheless, there was still one isolate in query. More informative genes such as genes in the mating-type locus are required to resolve the relationship between the unknown isolate and other species of FGSC (O’Donnell et al. 2004).

*Fusarium asiaticum* was identified as a predominate species with 98% of the population in this study. *Fusarium asiaticum* and *F. graminearum* s. str. were the major species in many Asian countries (van der Lee et al. 2015; Wang et al. 2011). Factors like temperatures and hosts have been ascribed to species distribution. In China, *F. asiaticum* was mainly distributed in the southern provinces, whereas *F. graminearum* s. str. was mostly in the northern provinces (Qu et al. 2008b; Shen et al. 2012). The same trend was observed in an analysis of Japanese isolates (Suga et al. 2008). From a study of FHB of wheat, Qu et al. (2008b) concluded that *F. asiaticum* was mainly obtained from warmer regions where the annual average temperature were above 15 °C, and the majority of *F. graminearum* s. str. was isolated from cooler regions with the annual average temperature at 15 °C or lower. A BIOCLIM analysis suggested that areas where the warmest quarter with the mean temperature above 22 °C and the precipitation over 320 nm favored the occurrence of *F. asiaticum* (Backhouse 2014). In addition, the host preference was implicated by a study of rice population in South Korea in which *F. asiaticum* (FGSC lineage 6) dominated over *F. graminearum* s. str. (FGSC lineage 7) and *F. boothii* (FGSC lineage 3) (Lee et al. 2009). The study showed that *F. asiaticum* produced more perithecia on rice straw than the other two species in an experiment of mixed-species inoculum. Zhang et al. (2012) found a strong association between the predominate crops and the occurrence of *F. asiaticum* and *F. graminearum* s. str. They showed that *F. asiaticum* was more frequently obtained from the middle and low valleys of Yangtze River (southern China) where rice acreages were higher than maize acreages. *Fusarium graminearum* s. str. was prevalent in northern China with higher maize acreages (Zhang et al. 2012). In our study, the average annual temperatures of sampling areas in Taiwan were 22 °C and higher, and the precipitation of the warmest quarter were from 322 to 608 nm. All areas where samples were collected were operated under the rice–wheat rotation system. The result of predominate *F. asiaticum* in Taiwan was consistent with the notion of those previous studies.

Fg16F and Fg16R were designed as specific primers for FGSC isolates (Nicholson et al. 1998). The various amplicons from Fg16F/R PCR were used for the SCAR analysis of FGSC isolates to reveal the genetic variation (Carter et al. 2000, 2002; Qu et al. 2008a, b; Desjardins et al. 2004). Later studies showed that SCAR type 1 and type 5 were fully congruent with *F. graminearum* s. str. and *F. asiaticum*, respectively, and were used to screen these 2 phylogenetic species (Chandler et al. 2003; Qiu et al. 2014; Zhang et al. 2007). SCAR type 2 was congruent with *F. meridionale*, but contained the same PCR band size as SCAR type 5 (Chandler et al. 2003). In this study, isolates of SCAR type 1 and most SCAR type 5 were confirmed as *F. graminearum* s. str. and *F. asiaticum*, respectively, by PCR–RFLP and phylogenetic analysis. Some *F. asiaticum* isolates were of SCAR type 3 and SCAR type 4, which has been reported elsewhere (Chandler et al. 2003). Notably, the one *F. meridionale* isolate (Fanyuan1-11) was determined to be SCAR type 5, rather than SCAR type 2. The unknown species isolate (Daya272-3) was of SCAR type 2, but was phylogenetically closest to *F. cortaderiae* rather than *F. meridionale*. These results suggested that additional caution was needed to be taken to infer FGSC species by SACR types.

It was surprising to find that *F. asiaticum* isolates were either 15-ADON or NIV genotype in this Taiwanese population. The 3-ADON genotype isolate was not detected in current collection. The 15-ADON genotype isolates were more prevalent than NIV genotype isolates in all geographic areas, especially in northern Taiwan where 15-ADON genotype isolates accounted for 99% of the population. In Japanese and Chinese populations of *F. asiaticum,* all 3 trichothecene genotypes (3-ADON, 15-ADON and NIV genotypes) were detected. Both 3-ADON and NIV genotype isolates usually dominated over 15-ADON genotype isolates (Qiu et al. 2014; Suga et al. 2008). Outside of Asia, NIV genotype isolates of *F. asiaticum* were prevalent in southern Louisiana, United States and southern Brazil (Gale et al. 2011; Gomes et al. 2015). The 15-ADON genotype isolates of *F. asiaticum* were a typical minority and may be absent in culture collections (Karugia et al. 2009; Shen et al. 2012). In China, the 3-ADON and NIV genotype isolates were prevalent in the warmer southern regions whereas the 15-ADON genotype was mainly presented in the cooler northern regions. The geographic difference of trichothecene genotypes were explained by the unevenly distribution of phylogenetic species. *F. asiaticum*, the warmer-region inhabitant, mainly belonged to 3-ADON and NIV genotypes based on 1903 isolates while *F. graminearum* s. str., the cooler-region inhabitant, contained more 15-ADON genotype isolates based on 383 isolates, according to several studies in China and Japan (Karugia et al. 2009; Qiu et al. 2014; Shen et al. 2012; Suga et al. 2008; Zhang et al. 2007). Regarding the geographic locations of 15-ADON genotype isolates which were mostly in the north of East Asia, this Taiwanese *F. asiaticum* 15-ADON isolates represented a unique population locating in the south of East Asia. This result suggested that 3-ADON genotype isolates were possibly not existed in Taiwanese populations or several factors may favor the 15-ADON genotype isolates to establish in Taiwan. It is notable that the wheat cultivar Taichung Sel. 2 derived from the wheat line Au-Maya74“S” was the only wheat cultivar used for wheat production in Taiwan currently. All FGSC isolates were isolated from diseased plants of the cultivar. The pathogenicity assay, however, indicated that the host cultivar was equally susceptible to 15-ADON and NIV genotype isolates. It needed further evaluations if the Taichung Sel. 2 cultivar favors *F. asiaticum* 15-ADON genotype isolates for growth, survival, sporulation, or other biological traits that would facilitate these 15-ADON genotype isolates to outcompete other genotype isolates.

## Conclusions

This study was the first report on phylogenetic species and trichothecene genotypes for a systematically collected FGSC population in Taiwan. *Fusarium graminearum* s. str., *F. meridionale*, *F. asiaticum*, and an unknown species were identified, and both *F. meridionale* and *F. asiaticum* were first reported in Taiwan. Of 195 isolates, *F. asiaticum* (n = 192) was the predominate species of FGSC wheat isolates and prevalent in northern and central Taiwan. Most Taiwanese *F. asiaticum* isolates contained 15-ADON trichothecene genotype (n = 162), and the rest of them contained NIV trichothecene genotype (n = 33). The high 15-ADON genotype of *F. asiaticum* population in Taiwan was a sharp contrast to the high 3-ADON genotype of *F. asiaticum* reported in the neighbor countries. The above information also provided valuable information for the global distribution of FGSC in East Asia (van der Lee et al. 2015).

## Additional file

**Additional file 1: Figure S1.** The morphology of FGSC species with different SCAR types and genotypes. **a** An unknown species (Daya 272-3). **b** *F. meridionale* (Fanyuan 1-11). **c** *F. graninearum* s.str. (Daya 211-13). **d** *F. asiaticum* SCAR type 3 DON (Daya 350-11). **e** *F. asiaticum* SCAR type 4 NIV (Daya 350-2). **f** *F. asiaticum* SCAR type 5 NIV (Daya 350-5). **g** *F. asiaticum* SCAR type 4 DON (K-r-1). **h** *F. asiaticum* SCAR type 5 DON (R-p-1). The column-1 and column-2 were the 10-day PDA cultures. The column-3 was the 7-day CLA culture. The column-4 and column-5 showed the perithecia and ascospores, respectively. The column-6 showed macroconidia produced on CLA cultures (scale bar = 10 μm).

## Abbreviations

FHB: Fusarium head blight
FGSC: *Fusarium graminearum* species complex
SCAR: sequence characterized amplified region
RFLP: restriction fragment length polymorphism
DON: deoxynivalenol
15-ADON: 15-acetyl-deoxynivalenol
3-ADON: 3-acetyl-deoxynivalenol
NIV: nivalenol
PDA: potato dextrose agar
CLA: carnation leaf agar
