# Supplementary figures and images for: Identification and trichothecene genotypes of Fusarium graminearum species complex from wheat in Taiwan
Source: Bot Stud. 2017 Jan 2;58:4. doi: 10.1186/s40529-016-0156-4 (PMC5430562; doi:10.1186/s40529-016-0156-4)

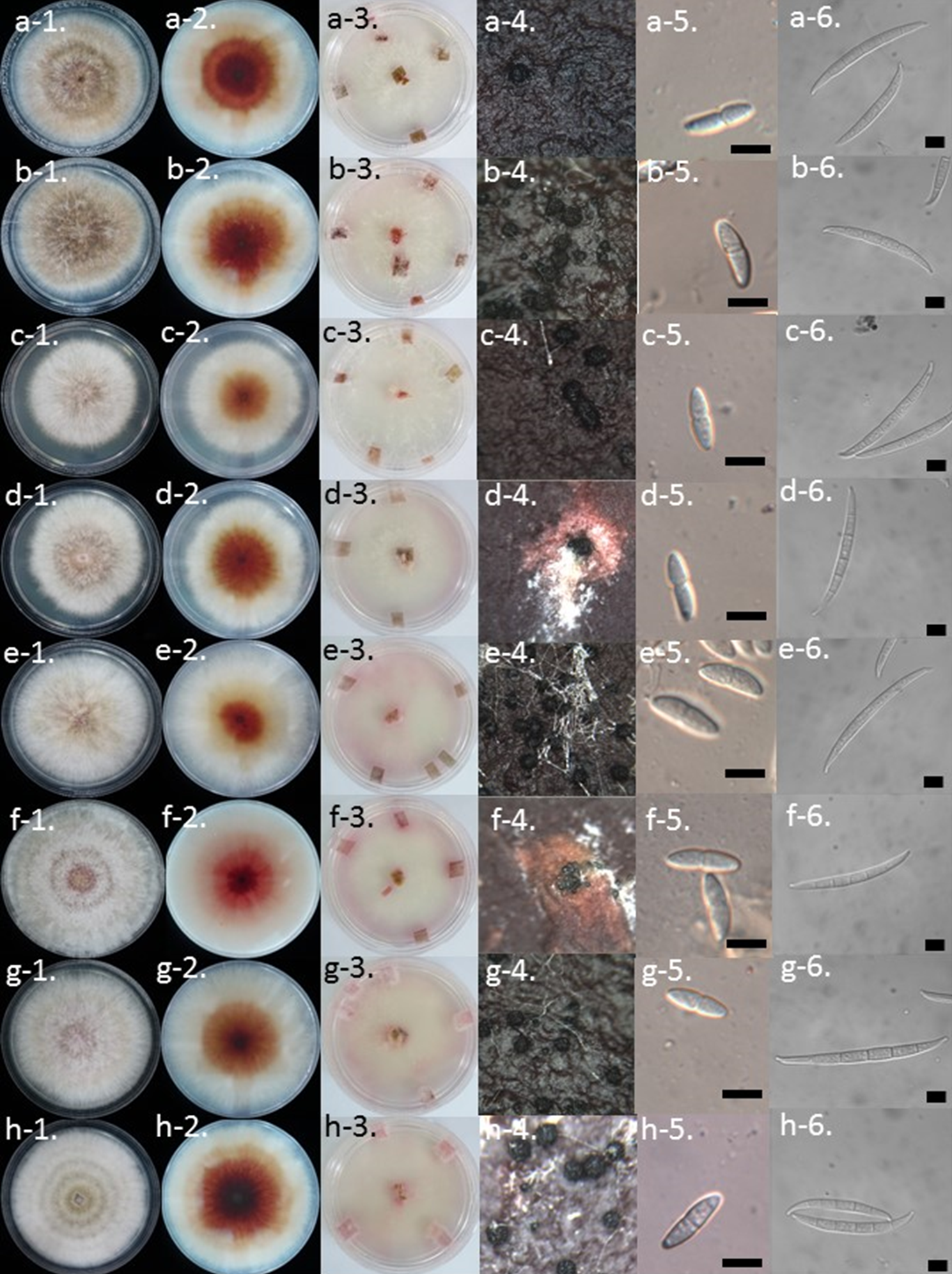

Supplement: Supplementary file 1 — Additional file 1: Figure S1. The morphology of FGSC species with different SCAR types and genotypes. a An unknown species (Daya 272-3). b F. meridionale (Fanyuan 1-11). c F. graninearum s.str. (Daya 211-13). d F. asiaticum SCAR type 3 DON (Daya 350-11). e F. asiaticum SCAR type 4 NIV (Daya 350-2). f F. asiaticum SCAR type 5 NIV (Daya 350-5). g F. asiaticum SCAR type 4 DON (K-r-1). h F. asiaticum SCAR type 5 DON (R-p-1). The column-1 and column-2 were the 10-day PDA cultures. The column-3 was the 7-day CLA culture. The column-4 and column-5 showed the perithecia and ascospores, respectively. The column-6 showed macroconidia produced on CLA cultures (scale bar = 10 μm). [file 40529_2016_156_MOESM1_ESM.tif]
